# Targeting inflammatory pathways in axial spondyloarthritis

**DOI:** 10.1186/s13075-019-1885-z

**Published:** 2019-06-04

**Authors:** Daniel E. Furst, James S. Louie

**Affiliations:** Department of Medicine, Division of Rheumatology, David Geffen School of Medicine at UCLA, Los Angeles, CA USA

**Keywords:** Axial spondyloarthritis, Therapy, Inflammatory pathways

## Abstract

The triggers and pathogenesis of axial spondyloarthritis (axSpA) are not yet completely understood. However, therapeutic agents targeting tumor necrosis factor-α and interleukin-17 inflammatory pathways have proven successful in suppressing many of the clinical symptoms and signs of axSpA, giving us an indication of which pathways are responsible for initiating and maintaining the inflammation. The mechanisms that eventuate in syndesmophytes and ankyloses are less clear. This review addresses these two critical pathways of inflammation, discussing their nature and these factors that may activate or enhance the pathways in patients with axSpA. In addition, genetic and other markers important to the inflammatory pathways implicated in axSpA are explored, and prognostic biomarkers are discussed. Treatment options available for the management of axSpA and their associated targets are highlighted.

## Background

Axial spondyloarthritis (axSpA) is the prototypical form of a family of diseases known as spondyloarthritis (SpA) characterized by inflammatory processes and new bone formation [1, 2]. Inflammation, bone and cartilage loss, and subsequent remodeling with new bone formation take place in the entheses, axial skeleton, and peripheral joints. Critically, axSpA encompasses two conditions: ankylosing spondylitis (AS), which presents with radiographic damage and ankyloses of the sacroiliac joint (as defined and validated by the Assessment of SpondyloArthritis international Society [ASAS] classification criteria) [3, 4], and nonradiographic axSpA (nr-axSpA), which does not show radiographic changes but may describe inflammation to the sacroiliac joint by magnetic resonance imaging (MRI), power Doppler ultrasound (PDUS), or computed tomography [5, 6]. The prevalence of axSpA in the USA is estimated to be 0.7%, with AS and nr-axSpA each accounting for 0.35% of patients [7]. Patients with axSpA also present with a range of extra-articular manifestations, including inflammatory bowel lesions, psoriasis, and uveitis [1, 8, 9]. Thus, axSpA is a potentially debilitating disease, associated with chronic pain, deformities, and reduced function and quality of life [10, 11].

The pathogenesis of axSpA appears to be multifactorial, arising from several exogenous factors, engaging genetic susceptibilities to amplify multiple inflammatory and innate and acquired immune responses, and eventuating in musculoskeletal damage and repair. Clinically, the initiating factors include biomechanical stresses affecting the tissues and cells of the entheses, where tendons and ligaments bind to the fibrocartilage and bone. McGonagle and colleagues [12] substantiated the concepts of tissue micro-damage, whereby stresses in the synovial–entheseal complex trigger interleukin (IL)-23 from macrophages, dendritic cells, and possibly group 3 innate lymphoid cells (ILC3s) to initiate inflammation in the adjoining fibrocartilage, bursae, fat pad, deep fascia, synovium, and cortical and trabecular bone. The most stressed regions occur in the sacroiliac, spinal, sternoclavicular, manubriosternal, and acromioclavicular joints, rather than in peripheral joints [13, 14].

Another critical initiating factor may come from infectious signals generated from commensal bacteria within the gut microbiome, which moderate immune homeostasis of the innate and innate-like cells at the barrier sites [15, 16]. In axSpA, innate mesenchymal stem cells, monocytes, and dendritic cells permit expression of IL-23 receptor-positive ILCs in the gut, blood, synovial fluid, and bone marrow, and IL-23–positive cells in spinal facet joints, which activate the IL-23/IL-17 axis of pro-inflammatory cytokines [17, 18]. In addition, the mechanisms that activate monocytes with lipopolysaccharides (LPS) and other bacterial adjuvants and recruit neutrophils to the entheses may be strong contributing factors to both the production of tumor necrosis factor-α (TNF) and the activation of osteoblasts [19]. Interestingly, increased levels of monocyte/macrophage migration inhibition factor (MIF) in peripheral blood correlate with disease activity and predict spinal syndesmophyte progression [20]. Furthermore, elevated autoantibodies to the CD74 receptor for MIF are considered to be a diagnostic marker for axSpA, even in patients who do not express human leukocyte antigen (HLA)-B27 [21].

Susceptibility to axSpA in people with the *HLA-B27* and *HLA-B40* genes has been elegantly proposed; however, clear and consistent agreement across studies is lacking. It is not clear whether HLA and non-HLA genes and polymorphisms of the *IL23R* gene permit a lower threshold of mechanical stress or LPS levels to be activated, although increased gut permeability has been proposed. In addition, the chronic nature of the inflammatory immune responses in axSpA may be due to aberrant peptide processing and presentation, sustained triggering of inflammatory pathways, and failure of inflammation to resolve in these HLA-B27 and HLA-B40 genetically predisposed individuals [9, 22]. Furthermore, what triggers and maintains new bone formation and ankyloses in axSpA is not fully understood, and it is not clear which therapeutic modalities can clearly arrest the deformities caused by new bone formation.

It is strongly suggested that the earliest therapies to forestall inflammation will restrict damage and subsequent bone formation and ankyloses and thus allow patients to maintain function and quality of life. The latest recommendations reinforce the concept of treating towards defined and validated measures of disease activity, as assessed by the Ankylosing Spondylitis Disease Activity Score (ASDAS), or the Bath Ankylosing Spondylitis Disease Activity Index (BASDAI), recording improvement based on achievement of ASAS20 or ASAS40, and changing therapies if ASDAS scores do not indicate remission (i.e., scores < 1.3) or ASAS partial remission scores do not decrease by at least two units on a 0-to-10 scale in four domains. Secondly, clinical practice has validated that therapeutic successes depend on the educated patient who has committed to mutually agreed-upon goals with the rheumatologist, who regularly communicates the clinical data [23–25].

## Methods

Targeted PubMed literature searches were conducted to identify articles that discussed inflammatory pathways and genes involved in the development of axSpA. Searches were conducted using combinations of search terms, including “ankylosing spondylitis,” “axial spondyloarthritis,” “inflammation,” “pathway,” “pathogenesis,” “gene,” “biomarker,” “polymorphism,” “bone formation,” “bone loss,” “comorbidities,” “IL-1,” “IL-6,” “IL-17,” “IL-23,” and “TNF/tumor necrosis factor.” Search results were supplemented based on the reference citations in articles identified in initial searches and based on the authors’ familiarity with the published literature. Articles were qualitatively selected for inclusion in this review if they presented results that the authors deemed relevant.

## Therapies

The mainstay of pharmacologic treatment for both AS and nr-axSpA begins with nonsteroidal anti-inflammatory drugs (NSAIDs) [26], which inhibit the cyclooxygenase (COX) activity of prostaglandin E2 (PGE2). PGE2 initiates inflammation by activating macrophages, mast cells, neutrophils, and site-specific stromal and vascular endothelial cells and facilitates the transition from innate to acquired immune responses by enhancing the IL-23/IL-17 axis and developing the regulatory T cell. Specifically, PGE2 acts on T-helper (Th)1 and Th17 cells via its EP2 and EP4 receptors in the presence of IL-1β and IL-23; receptor polymorphisms may affect the efficacy of COX inhibitors in axSpA [27]. Inhibiting PGE2 resolves entheseal inflammation, relieves pain, inhibits vasodilation, and retards bone formation, particularly if used continuously rather than intermittently, as confirmed by ultrasound and x-rays [28, 29]. Thus, NSAID therapies are strongly recommended [23, 25, 30].

The traditional disease-modifying anti-rheumatic drugs (DMARDs), such as methotrexate, leflunomide, and sulfasalazine, were not found to be effective in controlling AS or nr-axSpA [26, 31]. However, analysis of data from the Swedish Biologics Register showed that the combination of conventional synthetic DMARDs (especially methotrexate) with TNF inhibition enhances retention to anti-TNF therapy [32].

Data from clinical trials have described that inhibitors of IL-1 [33] and IL-6 [34], as well as therapy with abatacept [35, 36] and rituximab (CD20) [37], did not appear to be useful in AS. Rather, the current therapeutic approach that is strongly recommended for the treatment of axSpA (regardless of whether or not radiographic disease is present) [26] centers on the use of biologic treatments directed at more precise cytokine targets, including TNFα [11, 38–40] and IL-23/IL-17 [41, 42]. Thus, we will address first the TNF and IL-17 pathways, including the nature of the pathways, and the factors that may activate or enhance the pathways in patients with axSpA or in animal models or in vitro experiments. Second, genetic and other markers important to the initial and then continuing inflammatory pathways implicated in axSpA will be described, including the sparse emerging data on prognostic biomarkers. Finally, the range of treatment options available for the management of axSpA and their associated targets will be explored.

## The inflammatory process: role of TNF and IL-17/IL-23

Cytokines produced by various cells play an important role in driving the immune response in axSpA and other inflammatory arthritic diseases. Advances in molecular and immunologic research over the past three decades have repeatedly supported the key role of cytokine dysregulation in the pathophysiology of auto-inflammatory and autoimmune diseases, including axSpA [9].

### TNF

**Fig. 1:**
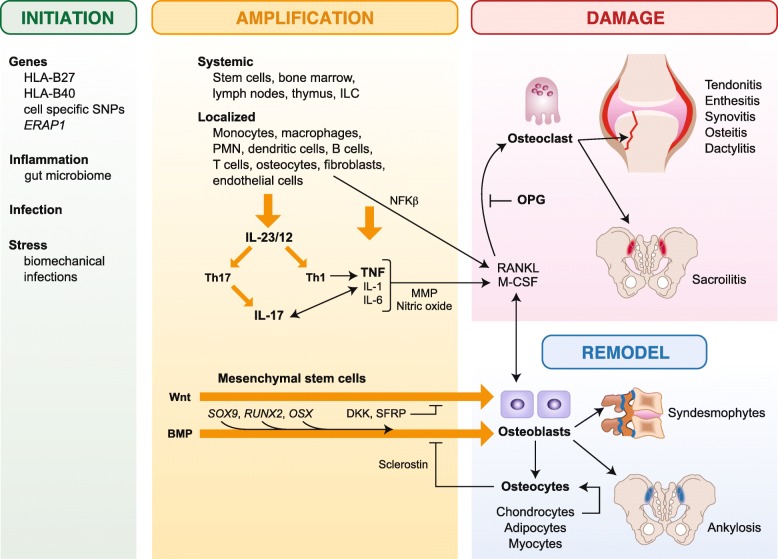
Inflammatory Pathways in axSpA. In spondyloarthritis, biomechanical stress and inflammatory factors, including infectious antigens, amplified by MHC susceptibility genes, HLA-B27 variants, and ERAP1 SNP transcription factors induce specific cell types to produce a series of inflammatory cytokines, including IL-23, IL-17, TNF, IL-1, and IL-6. Hematopoietic stem cells elaborate NF-κB, RANKL, and M-CSF to differentiate monocytes to osteoclasts, which extend inflammatory damage in the supporting structures of the sacroiliac and peripheral joints. Mesenchymal stem cells facilitated by Wnt and BMP differentiate to osteoblasts to form new bone and ankyloses. BMP, bone morphogenetic protein; CRP, C-reactive protein; DKK, Dickkopf; HLA, human leukocyte antigen; *ERAP1*, endoplasmic reticulum aminopeptidase 1; IL, interleukin; ILC-3s, group 3 innate lymphoid cells; M-CSF, monocyte colony-stimulating factor; MMP, metalloproteinase; *NF*-κB, nuclear factor-κB; OPG, osteoprotegerin; *OSX*, osterix zinc finger-containing transcription factor; RANKL, receptor activator of nuclear factor-κB ligand; *RUNX*, runt-related transcription factor; *SFRP*, secreted frizzled-related proteins; SNP, single-nucleotide polymorphism; SOX9, sex-determining region Y transcription factor; TNF, tumor necrosis factor; VEGF, vascular endothelial growth factor. Italicized = transcription factor; red = inflammation; blue = bone formation/ankylosis. Th1 cells = T helper cells for humoral immunity; Th17 cells = T helper cells for IL-17 inflammation, damage

Several lines of evidence implicate TNF in the pathogenesis of axSpA; however, the exact TNF-associated cellular and molecular mechanisms involved are not well understood [2]. TNF, a 233 amino acid protein, is synthesized as a transmembrane protein (tmTNF), in a trimeric form which can be cleaved by a converting enzyme to a soluble molecule (sTNF). Both act by binding to two receptors, tumor necrosis factor receptor (TNFR) 1 and TNFR2, activating multiple cellular pathways ranging from cell homeostasis and proliferation via nuclear factor (NF)-κB and protective immunity from infections and cell death via the caspases, respectively (Fig. 1) [2]. Additionally, tmTNF can act as a ligand by binding cell-to-cell to TNF receptors and as a receptor that transmits reverse or outside-to-inside signals to induce local inflammation within the tmTNF-bearing cells. [43].

Key TNF signaling pathways include TNFR1 or TNFR superfamily type 1A (TNFRSF1A), TNFR1-associated death domain (TRADD), and TNFSF15 [1, 2, 44]. TNFR1 signaling is associated with NF-κB–mediated cell survival and growth and/or apoptosis through the TRADD adaptor protein [2]. TNFR2 is predominantly produced by cells of immunologic and endothelial origin, and is also associated with NF-κB activation [2, 45]. Overexpression of TNF has been documented in the sacroiliac joints of patients with AS, and patients with AS have been shown to have high levels of the circulating soluble TNF receptors (sTNF-R1 and sTNF-R2) [46].

### IL-23/IL-17 axis

Immunologic studies have strongly implicated the IL-23/IL-17 axis in axSpA [2, 9]. Macrophages, dendritic cells, ILCs, and mucosal-associated invariant T cells (MAITs) contribute to elevated IL-23 levels found in the serum, inflamed gut, synovial fluid, entheses, and bone-derived cells, including the marrow, and fibrous tissues taken from the peripheral joints of patients with AS [47–52]. In addition, IL-17–positive mast cells and neutrophils have been reported in facet joints [53], and IL-22 levels have been shown to be elevated in the gut of patients with AS [54]. Macrophages from patients with AS stimulated by Toll-like receptor agonists such as LPS produced IL-23; and IL-23 enhanced IL-17 production from pathogenic Th17 cells [55].

In response to IL-23, additional cells, including CD4^+^ and CD8^+^ T cells in the microenvironment of other pro-inflammatory cytokines, chemokines, and transcriptional regulators (e.g., signal transducer and activation of transcription [STAT] 3, RAR-related orphan receptor γt [RORγt]), drive Th17 cells to produce IL-17 and other cytokines, including IL-6, IL-22, IL-26, and TNF in a reciprocating continuum [9, 56, 57]. The gut microbiome may also contribute, as serum amyloid A from the terminal ileum induces Th17 cell differentiation from naïve CD4^+^ T lymphocytes, and microbiota-induced IL-1β stimulates development of Th17 lymphocytes in the intestine [58].

Some have proposed that HLA-B27 misfolding and homodimer formation triggers IL-23 and IL-17 production through unfolded protein response and autophagy, further supporting the link between the IL-23/IL-17 pathway and the development of axSpA [2, 59]. This supports the emerging role of a pathogenic IL-23/IL-17 axis in axSpA [55, 60].

## Genes and cytokines associated with axSpA

Extensive genome-wide association studies (GWAS) and other studies have identified genes involved in the development of axSpA, providing further insight into potential therapeutic and diagnostic targets (Table 1) [2, 22, 61–63]. Axial spondyloarthritis appears to have high heritability, not only in terms of susceptibility but also in determining disease severity and functional incapacity [64]. The gene-encoding HLA-B27, found within the major histocompatibility region, is the most well-established genetic marker for axSpA [65–67]. Approximately 90% to 95% of Caucasian patients with axSpA are positive for *HLA-B27*, compared with only 6% to 8% of the general population [68–71]. Wide ethnic variability in *HLA-B27* expression exists in patients with SpA [72]. Although the canonical function of HLA-B27 is to present antigens to CD8^+^ T cells, much of the altered innate immune response in SpA may be related to cytoplasmic functions, which prepare the peptide for effective presentation [1]. Thus, changes in the ubiquitination process, which direct proteins to the subcellular proteasome and aminopeptidase trimming of peptides, are important processes that are associated with axSpA [73]. Indeed, endoplasmic reticulum aminopeptidase (*ERAP)1* and *ERAP2* have also been associated with axSpA, with *ERAP1* conferring a relative attributable risk to AS of approximately 25% [74].

**Table 1:** Genes and gene polymorphisms linked with axSpA

Gene	Name	Pathway and/or putative function	References
Genes and gene polymorphisms			
*HLA-B27* *B2702, 2703, 2704, 2705, 2707, 2708, 2710, 2714, 2715, 2719; 2706 and 2709 (reduced risk)*	Human leukocyte antigen B27 105 subtypes encoded by 132 alleles; numerous genetic risk variants	Peptide presentation to T cells, HLA-B27 molecule misfolding leading to endoplasmic reticulum stress reaction, homodimer formation leading to natural killer (NK) cell activation Interacts with ERAP1	Caffrey 1973 [141] Brewerton 1973 [65] Schlosstein 1973 [66] Montserrat 2006 [142] Cipriani 2003 [143] Yang 2014 [144] Fiorillo 2003 [145] Khan 2013 [146] Jaakkola 2006 [147] Armas 1999 [148] Reveille 2006 [149]
*HLA-B40, B13, B47, B51 B60, B14*	Human leukocyte antigen B	Antigen recognition Interacts with ERAP1	Cortes 2015 [150] Van Gaalen 2013 [151]
Tumor necrosis factor (TNF) pathway			
*TNFRSF1A/TNFR1* (383 A/C, rs4149577, rs4149576, rs1860545, and rs7954567 polymorphisms)	Tumor necrosis factor receptor superfamily member 1A *(tumor necrosis factor receptor 1)*	TNF signaling Nuclear factor (NF)-κB activation and cytokine production	Corona-Sanchez 2012 [152] Davidson 2011 [153] Karaderi 2012 [154] Evans 2011 [22]
*TRADD* region on chromosome 16	*Tumor necrosis factor receptor type 1-associated death domain*	TNF signaling NF-κB activation and cell death	Pointon 2010 [155] Hsu 1995 [156]
Interleukin 23 pathway			
*IL-23R* (multiple polymorphisms)	Interleukin-23 receptor gene Elevated in AS gut epithelium, from CD 4, γδ T, NK, innate lymphoid, mast cells	Th17-mediated immunity Production of IL-17A, IL-17F, IL-22, and IFN-γ	Reveille 2010 [62] Burton 2007 [61] Danoy 2010 [157] Dong 2013 [158] Cortes 2013 [159] Di Cesare 2009 [160]
*IL12B* (rs6871626, rs10045431, and rs3212227 polymorphisms)	Interleukin-12B	Activation and differentiation of IL-23R-expressing cells	Danoy 2010 [157] Zhang 2015 [161] Wong 2012 [162]
*IL-6R* (rs4129267 polymorphism)	Interleukin-6R	Th17-mediated immunity TH17 cell differentiation	Reveille 2015 [75] Cortes 2013 [159]
*IL1R2* (rs2310173 polymorphism) *IL1R1-IL1R2* (rs4851529 and rs2192752 polymorphisms)	Interleukin 1 receptor, type I/II	Th17-mediated immunity Modulation of IL-1 response	Reveille 2010 [62] Reveille 2015 [75]
*JAK2* (rs10758669 polymorphism, rs1536798/rs10119004/rs7857730-CGT haplotype)	Janus kinase 2	IL-23R signaling molecule	Danoy 2010 [157] Chen 2010 [163]
*STAT3* (rs2293152, rs6503695, rs744166 polymorphisms)	Signal transducer and activator of transcription 3	IL-23R and IL-6 signaling molecule	Davidson 2011 [153] Danoy 2010 [157]
Lymphocyte development and activation			
*ERAP1* (multiple polymorphisms)	Endoplasmic reticulum aminopeptidase-1 Also pairs with Cw6 of psoriasis, B51 of Behcet’s, A29 of birdshot chorioretinopathy but not NOD2 of Crohn’s Puromycin-sensitive aminopeptidase	Peptide presentation-Interacts with HLA-B27 and HLA-B40	Reeves 2014 [164] Evans 2011 [22] Abdullah 2015 [165] Chen 2015 [166] Bang 2011 [167] Brown 2016 [168]
*TYK2* (rs35164067, rs6511701, rs280518 polymorphisms)	Tyrosine kinase 2	Signaling from cytokine receptors, including IL-23R	Reveille 2015 [75] Cortes 2013 [159]
*CARD9* (rs11145835, rs10781500 polymorphisms)	Caspase recruitment domain family, member 9	Development of Th17 activation to some pathogens	Ma 2014 [169] Evans 2011 [22]
*RUNX3* (rs6600247 polymorphism)	Runt-related transcription factor 3	Reduction in CD8 T cell counts	Reveille 2015 [75] Cortes 2013 [159]
*KIR3DL1*	Killer immunoglobulin-like receptor-3 DL1	Inhibits cytotoxicity of NK cells	Abdullah 2015 [165] Zvyagin 2010 [170]

One review identified more than 41 genes predisposing to AS [75]. A meta-analysis identified 905 differentially expressed genes in AS, which included 482 upregulated genes and 423 downregulated genes [76]. In particular, polymorphisms involved in the innate immune system (*CARD9*) and cytokine signaling pathways, including TNF, IL-1, IL-6, and IL-23/IL-17 axes, appear to be strongly associated with the development of SpA, as per their suggestive effect on Th17-mediated immunity [1, 2].

Epigenetics, the study of mechanisms that determine and perpetuate heritable genomic functions without alteration in the DNA sequence, add to the complexity of our understanding of the pathogenesis of axSpA. These changes may account for the heterogeneity of clinical features and response to targeted therapies observed across patient subgroups [77]. Epigenetics, including histone modifications, acetylation, methylation, phosphorylation, sumoylation, and microRNA, may also help to explain the influence of environmental risk factors on genetic variation and their contribution to phenotypic variation among patients with axSpA [77]. Although multiple studies have demonstrated that tissue-specific epigenetic modifications play a role in autoimmune diseases such as rheumatoid arthritis, data in axSpA remain to be described and validated [77, 78]. Indeed, because genes mostly function by interacting with each other and their actions are largely dependent on their cell and tissue context, continuing GWAS may require specific cell analyses correlating with imaging and biopsy data to discover pathogenetic effects.

## Biologic agents for the treatment of axSpA

The advent of biologic agents has expanded the axSpA treatment armamentarium. Treatment with biologic agents is recommended for patients with AS who have persistently high disease activity despite conventional treatments [79, 80]. Treatment with biologic agents is also recommended for patients with nr-axSpA, with the specification in the European Union that these drugs only be used in patients with objective signs of inflammation (e.g., elevated C-reactive protein [CRP] and/or inflammation of the sacroiliac joints or spine on MRI) [26].

### TNF blockade

The strongest evidence supporting the role of the TNF pathway in the pathophysiology of axSpA comes from the use of TNF inhibitors in the clinical setting. TNF inhibitors are effective in reducing pain and stiffness and improving function in patients with AS [81]. TNF inhibitors reduce inflammation in most patients, but may not provide long-term remission. Recent studies now demonstrate that TNF blockers slow spinal radiographic progression by reducing disease activity (e.g., based on changes in the modified Stoke Ankylosing Spondylitis Spine Score [mSASSS]) [82]. Complete inhibition of radiographic progression is possible, as evidenced by patients who have reached an inactive disease state, defined as ASDAS < 1.3; significant reduction in disease activity is defined as ASDAS < 2.1 [2, 81, 83, 84]. However, a reported 20% to 30% of patients with axSpA do not respond adequately to TNF inhibitors, resulting in the need for other treatment options [85].

For patients who do show an initial response to TNF inhibition, treatment persistence of 5 years has shown sustained efficacy and sustained benefits in spinal mobility, disease activity, physical function, and health-related quality of life [86, 87]. After 1 year of treatment with adalimumab, ASAS20 and ASAS40 responses were achieved by 82% and 62% of patients, respectively, and after 5 years of treatment with adalimumab, ASAS20 and ASAS40 clinical responses were achieved by 89% and 70% of patients, respectively [86]. Furthermore, adalimumab and etanercept increased spine and femoral neck bone mineral density of patients with active AS with low bone mineral density [88]. Interestingly, TNF blockade did not influence the IL-23/Th17 axis, but TNF blockade did significantly reduce the erythrocyte sedimentation rate and CRP levels [89].

TNF inhibitors approved for the treatment of AS include adalimumab, certolizumab pegol, etanercept, infliximab, and golimumab (Table 2). Adalimumab and etanercept are also approved for nr-axSpA in Europe, and certolizumab pegol is approved for nr-axSpA in the US and Europe. Comparative efficacy analyses indicate that all five of the approved TNF inhibitors provide comparable ASAS responses, with similar safety profiles [90]. Notably, the effects of different TNF inhibitors on the extra-articular manifestations of axSpA are not equivalent. For example, infliximab provides better improvement in acute anterior uveitis than etanercept and adalimumab, and etanercept is not an effective treatment for inflammatory bowel disease [85].

**Table 2:** Summary of licensed biologic agents indicated for the treatment of axSpA

Name	Mechanism of action	Indication	Administration	Pivotal study	Primary endpoint(s)	Safety considerations from prescribing information
Adalimumab [171]	Human IgG1k. Binds soluble and transmembrane TNF. All TNF monoclonal antibodies can lyse surface TNF-expressing cells in vitro in the presence of complement	US: AS EU: AS and nr-axSpA	40 mg every other week Half-life of ~ 14 days	ABILITY-1 [172]	ASAS40 at week 12 • Adalimumab: 36% (*P* < 0.001) • Placebo: 15%	• Serious infections • Invasive fungal infections • Malignancies • Anaphylaxis or serious allergic reactions • Hepatitis B virus reactivation • Demyelinating disease • Cytopenias, pancytopenia • Heart failure • Lupus-like syndrome
Certolizumab pegol [173]	Fab fragment of humanized anti-TNF fused to polyethylene glycol. Binds to human TNF-α. Cannot bind to Fc receptors, fix complement, or cross placenta	US: AS and nr-axSpA EU: AS and nr-axSpA	400 mg SC at 1, 2, and 4 weeks, then 200 mg q2w or 400 mg q4w Half-life of ~ 14 days	RAPID-axSpA [11]	ASAS20 at week 12 • Certolizumab 200 mg Q2W: 58% (*P* = 0.004) • Certolizumab 400 mg Q4W: 64% (*P* < 0.001) • Placebo: 38%	• Serious infections • Invasive fungal infections • Malignancies • Anaphylaxis or serious allergic reactions • Hepatitis B virus reactivation • Demyelinating disease • Cytopenias, pancytopenia • Heart failure • Lupus-like syndrome
Etanercept [174]	Fusion protein of extracellular-binding sites of 2 TNF p75 receptors linked to the Fc portion of human IgG1. Binds soluble TNF and lymphotoxin α (TNF-β) molecules	US: AS EU: AS and nr-axSpA	25 mg twice weekly Half-life of ~ 4 days	Double-blind randomized controlled trial [38]	ASAS20 at week 12 • Etanercept: 59% (*P* < 0.0001) • Placebo: 28% ASAS20 at week 24 • Etanercept: 57% (*P* < 0.0001) • Placebo: 22%	• Serious infections • Invasive fungal infections • Malignancies • Anaphylaxis or serious allergic reactions • Hepatitis B virus reactivation • Demyelinating disease • Cytopenias, pancytopenia • Heart failure • Lupus-like syndrome
Golimumab [175]	Human IgG1κ monoclonal antibody. Binds soluble and transmembrane human TNF-α	US: AS EU: AS	50 or 100 mg SC once/month Half-life of ~ 14 days	GO-RAISE [40]	ASAS20 at week 14 • Golimumab 50 mg: 59% (*P* < 0.001) • Golimumab 100 mg: 60% (*P* < 0.001) • Placebo: 22%	• Serious infections • Invasive fungal infections • Malignancies • Anaphylaxis or serious allergic reactions • Hepatitis B virus reactivation • Demyelinating disease • Cytopenias, pancytopenia • Heart failure • Lupus-like syndrome
Infliximab [176]	Chimeric mouse-human monoclonal antibody with human constant and murine variable regions. Binds with high affinity to soluble and transmembrane TNF-α	US: AS EU: AS	5 mg/kg at 0, 2 and 6 weeks, then every 6 weeks Half-life of ~ 9 days	ASSERT [39]	ASAS20 at week 24 • Infliximab: 61% (*P* < 0.001) • Placebo: 19%	• Serious infections • Invasive fungal infections • Malignancies • Anaphylaxis or serious allergic reactions • Hepatitis B virus reactivation • Demyelinating disease • Cytopenias, pancytopenia • Heart failure • Lupus-like syndrome • Hepatotoxicity • Cardiovascular and cerebrovascular reactions
Secukinumab [91]	Human anti-IL-17A monoclonal antibody	US: AS EU: AS	MEASURE 1: 10 mg/kg IV at weeks 0, 2, and 4 followed by 75 mg or 150 mg SC Q4W from week 8 MEASURE 2: 75 mg or 150 mg SC at 0, 1, 2, 3, and 4 weeks, then Q4W Half-life of ~ 27 days	MEASURE 1 [42] MEASURE 2 [42]	MEASURE 1: ASAS20 at week 16 • Secukinumab 75 mg: 60% (*P* < 0.001) • Secukinumab 150 mg: 61% (*P* < 0.001) • Placebo: 29% MEASURE 2: ASAS20 at week 16 • Secukinumab 75 mg: 41% (*P* = 0.10) • Secukinumab 150 mg: 61% (*P* < 0.001) • Placebo: 28%	• Serious infections • Inflammatory bowel disease • Anaphylaxis or serious allergic reactions

*q2w* every 2 weeks, *q4w* every 4 weeks, *IL*, interleukin, *nr-axSpA* non-radiographic axial spondyloarthritis, *SC* subcutaneously, *TNF* tumor necrosis factor

### IL-17 blockade

Results of clinical trials aimed at blocking the IL-23/IL-17 axis in patients with AS support involvement of this pathway in the pathogenesis of axSpA [2]. As such, IL-17 has emerged as a novel therapeutic target in AS. Secukinumab is the only IL-17A inhibitor currently indicated for the treatment of AS (Table 2) [41, 91]. Secukinumab demonstrated efficacy comparable to the TNF inhibitors clinically in a patient population that included both individuals with and without prior exposure to TNF inhibitors [41, 42]. Secukinumab (10 mg/kg) given intravenously at weeks 0, 2, and 4, then subcutaneously (150 mg or 75 mg) every 4 weeks was associated with ASAS20/40 response rates of 61%/42% compared with 29%/13% with placebo (*P* < 0.001 for both) at week 16 [42]. When secukinumab was given only subcutaneously, ASAS20/40 was achieved by 61%/36% and by 41%/26% of patients treated with secukinumab 150 mg and 75 mg, respectively, compared with 28%/11% with placebo [42]; responses were maintained at week 52 [92]. The efficacy of secukinumab has also been demonstrated over 2 years in patients who are TNF naïve (ASAS20/40 response rates at week 104 of 77%/56% with secukinumab 150 mg and 80%/60% with secukinumab 75 mg) [92]. Further, an observational study of treatment with secukinumab (2 × 10 mg/kg intravenous loading doses followed by 3 mg/kg intravenously every 4 weeks for 94 weeks) reported low progression of spinal radiographic changes with 87% of the inflammatory vertebral edges and 30% of vertebral edges with fatty lesions at baseline resolved by week 94 [93].

Ixekizumab, an IL-17 inhibitor in an IgG4 formulation, has been approved for psoriasis and psoriatic arthritis, and is under investigation for treatment of AS (NCT02696785). At 16 weeks, ixekizumab 80 mg every 2 or 4 weeks elicited ASAS 20/40 response rates of 69%/52% and 64%/48%, respectively, compared with 40%/18% with placebo (*P* < 0.01 for all) and 59%/36% with adalimumab. In addition, there were significant reductions in spinal inflammation measured by MRI Spondylitis Research Consortium of Canada (SPARCC) spine score change from baseline [94]. Studies of ixekizumab (NCT02696798) and the IL-17RA inhibitor brodalumab (NCT02985983) are ongoing in patients with axSpA.

The dual IL-17A and IL-17F inhibitor, bimekizumab, has also demonstrated therapeutic potential for the treatment of AS. In a phase 2b study, 12 weeks of treatment was associated with an ASAS40 response rate of up to 47% compared to 13% with placebo (*P* < 0.001) [95].

Of great interest would be the use of bispecific biologic therapies that inhibit both TNF and IL-17 [96]. Dual inhibition of these cytokines was more effective at suppressing arthritis in a collagen-induced arthritis model than TNF inhibition alone [97].

### IL-12/IL-23 blockade

Ustekinumab, a human IgG1κ monoclonal antibody that binds to the common p40 subunit of IL-12 and IL-23, failed to meet the primary endpoint of a phase 3 study in patients with AS naïve to TNF inhibitors (NCT02437162). At week 24, ASAS40 was achieved by 31% of patients receiving ustekinumab 45 mg, 28% of patients receiving ustekinumab 90 mg, and 28% of patients receiving placebo. Additional phase 3 studies of ustekinumab in AS refractory to TNF inhibitors (NCT02438787) and nr-axSpA (NCT02407223) were terminated based on this result [98].

Similarly, risankizumab, a humanized IgG1 monoclonal antibody specific for the IL-23 p19 subunit, failed to meet the primary endpoint of a phase 2 study in biologic naïve patients with AS [99]. At week 12, ASAS40 response rates were 25% with risankizumab 18 mg, 21% with risankizumab 90 mg, 15% with risankizumab 180 mg, and 18% with placebo. Tildrakizumab, another IL-23 p19 monoclonal antibody, is in development for axSpA (NCT02980705). There are no currently planned studies of the IL-23 p19 monoclonal antibody, guselkumab, in AS.

The negative results from these studies of ustekinumab and risankizumab were surprising and have led to speculation about the differing biologic mechanisms that occur with IL-23 versus IL-17 inhibition. There may be pathogenic differences between inflammation at spinal and peripheral sites, possibly related to different mechanical load and stress responses of ligament and tendon insertions through PGE2 activation of ILC3s and production of IL-17 via an IL-23–independent pathway [100]. Of particular interest, recent studies using single-cell–based technology found that ILC3s were not a significant source of IL-17 in the joints of patients with peripheral SpA [101, 102]. It has also been hypothesized that IL-23 may play a pathogenic role in only certain stages of axSpA (e.g., during initiation but not established disease), suggesting that higher serum concentrations of IL-23 inhibitors may be necessary to have clinically meaningful effects [100].

### JAK blockade

The Janus kinase (JAK)/STAT pathway is thought to activate the IL-23/IL-17 cytokine axis, and inhibition of the JAK-STAT pathway has been proposed as a therapeutic strategy in AS [103]. In a phase 2 trial, the JAK 1/3 inhibitor tofacitinib demonstrated clinical efficacy in patients with AS [104]. At 12 weeks, tofacitinib 5 mg BID elicited ASAS20/40 response rates of 81%/46%; however, placebo responses were also high (41%/20%). Subanalysis discovered that the best responders were those with high CRP and higher MRI SPARCC scores. Indeed, the improvement in SPARCC sacroiliac joint and spine scores showed a dose response [104].

### IL-6 blockade

While serum IL-6 levels have been shown to be elevated in patients with AS, a recently published phase 2/3 study failed to demonstrate clinical benefit with tocilizumab, an IL-6 receptor–targeted monoclonal antibody, in TNF inhibitor-naïve patients with AS [34]. A related phase 3 trial in patients with AS who had an inadequate response to previous TNF inhibitor therapy was subsequently terminated (NCT01209689). A phase 2 study of sarilumab, an IL-6 inhibitor, in TNF inhibitor-naïve patients with active AS also failed to show a clinical benefit [105].

### IL-1 blockade

The IL-1 receptor family–specifically IL-1β–is a therapeutic target for several systemic and local auto-inflammatory conditions [106]. Two open-label studies have been conducted to investigate anakinra (an IL-1–receptor antagonist) in patients with AS. A pilot study suggested that anakinra may be effective in controlling AS symptoms; however, another study demonstrated limited improvement in only a small subgroup of patients with AS [33, 107].

### Phosphodiesterase 4 (PDE4) inhibition

A phase 3 randomized, double-blind study of apremilast, a selective inhibitor of PDE4, did not meet its primary endpoint of ASAS20 compared with placebo (32.5% vs 36.6%; *P* = 0.4383) in 490 patients with active AS [108].

## Mechanisms of bone formation and loss: Wnt pathway

Both cartilage and diffuse bone loss and pathologic new bone formation can be observed simultaneously in axSpA [2]. Mechanisms of inflammation are closely linked with bone metabolism and elevated levels of pro-inflammatory cytokines associated with bone loss [109]. Activated T lymphocytes and osteoblasts express receptor activator of NF-κB ligand (RANKL), a key regulator of bone remodeling via receptor activator of nuclear factor-κB (RANK)/RANKL/osteoprotegerin signaling, and both TNF and IL-1 induce RANKL resulting in bone loss [109]. Three other important pathways in bone remodeling are the wingless proteins/Dikkopf-1 (Wnt/DKK-1), secreted frizzled-related proteins and bone morphogenetic protein (BMP) pathways, which are affected by inflammation (Fig. 1) [109–111].

There is a dissociation of TNF-dependent inflammatory processes and TNF-independent bone-formation processes in axSpA, which may be triggered by mechanical and inflammatory stress [112, 113]. Radiographs are slow in documenting a decrease in new bone formation in patients with AS receiving TNF inhibitors when compared to historical controls [112, 114], and recent data with internal controls document less ankyloses with TNF inhibitor therapies [6]. MRI records decreases of bone marrow edema that lead to ankylosis [115] as anti-TNF therapies inhibit in vitro bone resorption by osteoclast precursor cells generated from peripheral blood with RANKL [116].

Indeed, TNF inhibitors may passively allow new bone formation to occur. TNF stimulates expression of DKK-1, which suppresses signaling by Wnt, a family of key mediators of osteoblast bone formation [117]. Inhibition of Wnt signaling by DKK promotes osteoblast and osteoclast formation and differentiation induced by BMP-2 [118]. These findings suggest that when anti-TNF therapies reduce inflammation, restoration of Wnt and BMP pathway signaling may occur, allowing the potential for new bone formation [2, 83, 119–121]. Additionally, therapeutic approaches are being investigated to activate the Wnt pathway, as antibodies targeting sclerostin and DKK-1 have shown promotion of bone formation and fracture healing for those with osteoporosis [122].

In animal models, IL-23R^+^ cells and RORγt^+^CD4^-^CD8^-^ T cells (which are responsive to IL-23) reside in the axial and peripheral entheseal interface between the tendon and bone [123–125]. Importantly, in B10.RIII mice, systemic expression of IL-23 sufficiently induced enthesitis in both the front and back paws without synovial joint destruction and promoted IL-17 and IL-22 expression by these entheseal cells. This process required recombinase activating gene (*Rag*)-dependent cells and occurred independently of Th17 cells, which is consistent with the primary role of IL-23 cells in enthesitis. Furthermore, systemic expression of IL-22 resulted in increased STAT3 phosphorylation in the bone and induced genes encoding Wnt family members, BMP, and alkaline phosphatase. Together these results indicate that the pathophysiology of enthesitis is mediated by IL-23 and its downstream targets, IL-17 and IL-22, whereas IL-22 is specifically involved in the osteoproliferation component of the disease [123].

## Next-generation clinical tools and biomarkers

ASAS classification and response criteria are valuable tools for most clinicians. However, they are based on patient-reported outcome measures, including pain, stiffness, fatigue, and patient global assessments, which can have high levels of both inter- and intra-individual variability and bias [126]. Thus, use of more objective measures should be considered in combination with ASAS criteria.

### Imaging

Imaging techniques, such as conventional radiography, bone scintigraphy, MRI, and PDUS, are used for the diagnosis of axSpA, monitoring disease activity, and assessing structural damage. Initially, the New York criteria for diagnosis of AS required radiographic evidence of sacroiliitis [127, 128]. Sacroiliitis appears only after several years of undiagnosed inflammatory back pain symptoms, while MRI has demonstrated that osteitis or bone marrow edema in the sacroiliac joint is present earlier—before it becomes radiographically detectable [127, 129]. Thus, MRI is increasingly being used to detect sacroiliitis early in patients with axSpA, particularly utilizing coronal and axial sequences and high-resolution erosion-specific sequences [127, 130–132].

MRI is useful for following therapeutic outcomes. Anti-TNF with infliximab therapy decreased spinal and sacroiliac osteitis scores more effectively than nonsteroidal therapies [133]. Similarly, anti-IL-17A therapy with secukinumab over 2 years decreased spinal osteitis scores and fatty lesions, which eventuate to ankyloses [93].

PDUS to identify increased vascular flow is also a highly sensitive and less costly tool for detecting enthesitis, which is not always detectable by clinical examination [134]. However, this method is limited by operator proficiency [125] and its use in sacroiliitis needs confirmation. In vivo probing of TNF for guidance of therapy using 99m Tc-labeled anti-TNF monoclonal antibodies and specific aptamers are proposed [135].

### Serum/tissue biomarkers

Investigations into the discovery of circulating and tissue-related biomarkers are ongoing. These biomarkers may help accurately diagnose axSpA, predict disease activity/progression, and improve response to therapy. The most frequently used axSpA marker in the clinical setting for diagnosis is HLA-B27 [136]. Erythrocyte sedimentation rate and CRP are not always dependable biomarkers for monitoring disease activity, but if elevated, predict better responses to biologic therapies and more comorbidities. [136]. Other research has focused on examining the prognostic value of cytokines, particularly IL-17 and IL-23, and downstream matrix metalloproteinase (MMP) markers such as MMP-3 (stromelysin-1), which degrades collagen II, III, IV, IX, X, and the extracellular matrix proteins. Markers of bony metabolism have also been investigated, including adipokines and cartilage/connective tissue degradation products [75]. However, no individual prognostic biomarker for disease activity has demonstrated adequate reproducibility, and an unmet need for robust biomarkers remains [75]. Biomarkers from the specific inflamed sites may be more informative and descriptive than from peripheral blood samples.

### Predictive markers

Various biomarkers have been investigated for their predictive value to treat axSpA. For example, in patients with axial and peripheral SpA, infliximab treatment response is associated with high-sensitivity CRP and calprotectin levels [137], while response to golimumab in patients with AS is associated with various combinations of markers comprising specific biomarker signatures [138]. Furthermore, secukinumab response after 6 weeks of treatment in patients with AS is associated with a decrease in levels of S100A8 and S100A9, which form calprotectin, the calcium-binding protein used as a marker of gut inflammation [41].

## Conclusions

As we learn about the complexities of the pathogenetic mechanisms that eventuate in axSpA, we cannot be surprised by the different clinical presentations and the variability of the individual responses to different therapies as the disease progresses over time. Each individual, possessed with different genetic susceptibilities, will undergo different initiating factors that will subject their specific cells to be activated by a varying milieu of pro-inflammatory and suppressive cytokines, chemokines, and transcriptional factors in different sites.

All agree that early diagnosis of axSpA is important because earlier treatment provides a more favorable prognosis, as irreversible structural damage occurs as the disease progresses. An early diagnosis of axSpA can also prevent the use of unnecessary diagnostic procedures and suboptimal treatments [139, 140].

Inhibition of TNF, IL-17, and other downstream cytokines and translational factors can reverse spinal inflammation. Careful clinical studies are required to differentiate if primary non-responders to either TNF or IL-17 inhibition will respond more effectively to other classes of therapy. Co-medication with conventional (NSAIDs) and newer downstream (JAK-STAT) therapies may be the next therapeutic recommendations to address the different treatment goals of controlling inflammation and subsequent bone damage [32]. Biomarkers recovered from the peripheral blood or from localized sites may predict susceptibility, activity, and clinical response to different therapies, but few are consistent and panels may be required. Accordingly, the current recommendations for changes to different therapies from knowledgeable investigators will be updated as clinical and translational studies continue. Thus, it will require careful clinical and imaging studies, especially in countries with a single medical care and documentation system, to differentiate whether any of the individual or combination therapies will modify bone damage and formation.

Finally, the rheumatologist undertakes the basic responsibility to educate and communicate, empowering the patient to be a committed partner in setting therapeutic goals, enabling the early referral of primary care physicians, and collaborating with the referring physician, therapist, primary care orthopedist, ophthalmologist, gastroenterologist, and other members of the healthcare team to promote exercise, smoking cessation, and high-quality continuing care. Thus, the rheumatologist who understands the significance of all of the clinical, imaging, genetic, and outcomes data is still the best decider of individual therapies.

## Abbreviations

AS: Ankylosing spondylitis
ASAS: Assessment of SpondyloArthritis International Society
ASDAS: Ankylosing Spondylitis Disease Activity Score
axSpA: Axial spondyloarthritis
BASDAI: Bath Ankylosing Spondylitis Disease Activity Index
BMP: Bone morphogenetic protein
COX: Cyclooxygenase
CRP: C-reactive protein
DKK-1: Dikkopf-1
DMARD: Disease-modifying anti-rheumatic drug
*ERAP*: Endoplasmic reticulum aminopeptidase
GWAS: Genome-wide association studies
HLA: Human leukocyte antigen
IL: Interleukin
ILC3: Group 3 innate lymphoid cells
JAK: Janus kinase
LPS: Lipopolysaccharide
MAIT: Mucosal-associated invariant T cells
MIF: Migration inhibition factor
MMP: Matrix metalloproteinase
MRI: Magnetic resonance imaging
mSASSS: Modified Stoke Ankylosing Spondylitis Spine Score
NF: Nuclear factor
nr-axSpA: Non-radiographic axial spondyloarthritis
NSAID: Nonsteroidal anti-inflammatory drug
PDE4: Phosphodiesterase 4
PDUS: Power Doppler ultrasound
PGE2: Prostaglandin E2
*Rag*: Recombinase activating gene
RANK: Receptor activator of nuclear factor-κB
RANKL: Receptor activator of nuclear factor-κB ligand
RORγt: RAR-related orphan receptor γt
SpA: Spondyloarthritis
SPARCC: Spondylitis Research Consortium of Canada
STAT: Signal transducer and activation of transcription
sTNF: Soluble tumor necrosis factor
sTNF-R: Soluble tumor necrosis factor receptor
Th: T-helper
tmTNF: Transmembrane tumor necrosis factor
TNF: Tumor necrosis factor-alpha
TNFR: Tumor necrosis factor receptor
TNFR1-TRADD: Tumor necrosis factor-associated death domain
TNFRSF1A: Tumor necrosis factor-receptor superfamily member type 1A
Wnt: Wingless protein
